# The differential toxicity of three different oxidized nickel compound nanoparticles and the effects of particle surface ligands in mouse alveolar macrophages

**DOI:** 10.1093/toxsci/kfaf133

**Published:** 2025-10-08

**Authors:** Rebekah L Kendall, Raymond F Hamilton, Jacob M Albright, Yu Zhao, Yingjie Hang, Chaoyun Tang, Dale Porter, Nianqiang Wu, Andrij Holian

**Affiliations:** Department of Biomedical and Pharmaceutical Sciences, Center for Environmental Health Sciences, University of Montana, Missoula, MT 59812, United States; Department of Biomedical and Pharmaceutical Sciences, Center for Environmental Health Sciences, University of Montana, Missoula, MT 59812, United States; Department of Biomedical and Pharmaceutical Sciences, Center for Environmental Health Sciences, University of Montana, Missoula, MT 59812, United States; Department of Chemical Engineering, University of Massachusetts Amherst, Amherst, MA 01003, United States; Department of Chemical Engineering, University of Massachusetts Amherst, Amherst, MA 01003, United States; Department of Chemical Engineering, University of Massachusetts Amherst, Amherst, MA 01003, United States; Pathology and Physiology Research Branch, Health Effects Laboratory Division, Centers for Disease Control and Prevention, National Institute for Occupational Safety and Health, Morgantown, WV 26505, United States; Department of Chemical Engineering, University of Massachusetts Amherst, Amherst, MA 01003, United States; Department of Biomedical and Pharmaceutical Sciences, Center for Environmental Health Sciences, University of Montana, Missoula, MT 59812, United States

**Keywords:** nanoparticle, nickel compound, nickel oxide, macrophage, LMP

## Abstract

Nickel-compound engineered nanomaterials (Ni-X NP) have diverse applications, yet their continued use raises concerns for potential health impacts upon exposure. This study investigated 3 structurally distinct Ni-X-NP—pure NiO (NCZ), NiO@Ni(OH)_2_ (SIG), and Ni@NiO@Ni(OH)_2_ (AA)—to determine how core composition and surface functionalization contribute to bioactivity. Each Ni-X NP was modified with surface moieties (–OH, –COOH, and –CH_3_) to assess the efficacy of surface modifications in reducing bioactivity. Ni-X NP were thoroughly characterized for structure, surface chemistry, and Ni^2+^ ion release in simulated lysosomal fluid. Red blood cells (RBCs) were used to evaluate the hemolytic capabilities of the nanoparticles, and primary murine alveolar macrophages (AM), and murine ex vivo alveolar macrophages (mexAM) were used to assess uptake, cytotoxicity, IL-1β release, and lysosomal membrane permeability (LMP). Results showed that NiO@Ni(OH)_2_ nanoparticles induced the greatest hemolysis in RBC, elicited the greatest IL-1β response in AM and mexAM, and produced the most LMP in mexAM. The Ni@NiO@Ni(OH)_2_ nanoparticle released the most Ni^2+^ and caused profound reductions in AM cell viability but failed to cause RBC hemolysis or LMP. Pure NiO nanoparticles exhibited minimal bioactivity and low Ni^2+^ release. Surface modification with (–COOH) or (–CH_3_) effectively reduced bioactivity in LMP-mediated inflammation but had minimal effect on Ni^2+^-driven toxicity. This study reveals that Ni-X NP bioactivity depends on both core composition and surface chemistry, and that surface functionalization reduces inflammation only when lysosomal damage is the primary driver. These findings underscore the need for careful design and evaluation of engineered nanomaterials.

Metal oxide-based engineered nanomaterials (ENM) have unique properties that originate with their small size and high surface-to-volume ratio and make them valuable for a variety of uses (Alhalili 2023). Nickel compound nanoparticles (Ni-X NP), including Ni, NiO, and Ni(OH)_2_ nanoparticles, are especially useful in that they are highly stable and have magnetic, electronic, optical, and catalytic properties that make them advantageous for use in a diverse array of applications ranging from biomedical applications to solar cells, catalysts, and wastewater purification (Farré et al. 2016; Berhe and Gebreslassie 2023; Umeh et al. 2025). In addition, Ni-X NP exhibit antibacterial and anticancer activity through the production of reactive oxygen species (ROS), leading to protein oxidation, DNA destruction, and apoptotic cell death (Gong et al. 2011; AlSalhi et al. 2020). Although these effects on cellular function make Ni-X NP valuable for medicine, they also have the potential to disrupt normal cell homeostasis and, if inhaled, contribute to pulmonary inflammation (Sager et al. 2016). Functionalization via physical or chemical attachment on Ni-X NP surface can influence dispersion, solubility, and stability. Moreover, it can tailor interactions with biological membranes and proteins, thereby broadening their utility in biomedical and therapeutic applications.

The rapid development of Ni-X NP has outpaced the evaluation of potential human health risks. It has been established that lung and systemic diseases can result from exposures to micron- or nano-sized particles (Furuya et al. 2018; Kirby 2019; The Lancet Respiratory Medicine 2019). There are growing concerns that the increased use of ENM, such as Ni-X NP, will add to the burden of lung and systemic diseases in humans exposed to inhalable particles in environmental and occupational settings (Powers et al. 2006; Braakhuis et al. 2014). Inhalation of nano-sized particles can trigger respiratory inflammation, primarily through interactions with alveolar macrophages (AMs) in the lungs (Hamilton et al. 2009; Nishi et al. 2020; Shimizu et al. 2023; Solorio-Rodriguez et al. 2023). AMs are the primary lung innate immune cells and take up inhaled particles as part of the first line of defense (Geiser and Kreyling 2010; You et al. 2020; Hetzel et al. 2021). Through phagocytosis, particles are taken up by AMs in cargo-carrying vesicles, phagosomes, and transported into the interior of AMs. Phagosomes then fuse with mature lysosomes to form phagolysosomes, where proteases strip particles of their protein/lipid corona (Wang et al. 2013; Cai et al. 2022). Most ENM, including Ni-X NP, are resistant to rapid (<4 h) dissolution (Shinohara et al. 2017) instead, they are free to interact with the phospholipid structures of the limiting phagolysosomal membrane (Wang et al. 2013; Alkhammash et al. 2015). These sustained interactions can facilitate phagolysosome membrane permeability (LMP), which results in the leakage of cathepsins and other lysosomal enzymes into the cytosol, thereby inducing an inflammatory response involving activation of the NLRP3 inflammasome complex and secretion of proinflammatory cytokines IL-1β and IL-18 (Thibodeau et al. 2004; Cassel et al. 2008; Dostert et al. 2008; Hornung et al. 2008; Rabolli et al. 2016; Sayan and Mossman 2016). In rats, intratracheal instillation of NiO nanoparticles resulted in persistent lung inflammation, driven by macrophage secretion of IL-1β in a caspase-1- and NLRP3-dependent manner (Cao et al. 2016), though the mechanisms through which NiO promotes inflammation are not well defined and focus largely on mitochondrial impacts.

Here, we used red blood cell (RBC), AM, and murine ex vivo alveolar macrophage (mexAM) to examine 3 Ni-X NPs in order to describe how changing surface properties that would affect subsequent interactions with biological surfaces would influence mechanisms that contribute to Ni-X NP-induced inflammation. We hypothesized that changing the surface properties of a series of Ni-X NPs would consistently decrease the bioactivity of the cells due to structure function relationships necessary for bioactivity. Three ligands that have been reported to alter NP surface charge were tested, –OH (weakly negative surface charge), –COOH (strongly negative surface charge), and –CH_3_ (neutral but can contribute to an overall weak positive surface charge). Raw and functionalized Ni-X NPs were physically characterized by evaluating size, morphology, and zeta potential. The release of nickel ions (Ni^2+^) in simulated lysosomal fluid (SLF) was measured, as well as the ability of the Ni-X NP to cause RBC hemolysis or initiate IL-1β release in AM. Cultured mexAMs were further utilized to confirm the ability of Ni-X NP to initiate IL-1β release and evaluate the potential of Ni-X NP to disrupt lysosome membranes and facilitate the release of cathepsin B from the lysosome.

## Materials and methods

### Materials

Raw nanoparticles were purchased from 3 suppliers. Nanochemazone (Cat #NCZ4701/0123A) (NCZ), Sigma-Aldrich (Cat #637130) (SIG), and Alfa Aesar (Cat #45505) (AA). These particles were characterized and subsequently functionalized with (–OH), (–COOH), and (–CH_3_) moieties. Tetramethylammonium hydroxide (TMAH) and toluene were purchased from Sigma-Aldrich. n-Butyltrimethoxysilane (BTMS) and 3-(triethoxysilyl)propylsuccinic anhydride (TEPSA) were purchased from Gelest Inc.

### Physical and chemical characterization of nanoparticles

Transmission electron microscopy (TEM) images were taken with a FEI Tecnai-T12 microscope (Philips, Holland). Scanning Electron Microscope (SEM) images were obtained by a FEI Magellan 400 XHR-SEM. Particle size distributions were statistically analyzed using ImageJ software. Dynamic light scattering and zeta potential characterizations were conducted in the dispersion media by the Malvern Zetasizer Nano ZSP system. X-ray diffraction (XRD) was tested by SAXSLAB GANESHA 300XL. X-ray photoelectron spectroscopy (XPS) characterization was taken by Thermo Scientific ESCALAB 250 Xi. Fourier Transform Infrared Spectrometer (FTIR) was measured by Thermo Fisher Scientific Nicolet iS50 FTIR Spectrometer.

### Functionalization of surfaces of Ni-X NP

Raw Ni-X NPs were modified with surface groups in a multi-step process. First, the –OH groups were added. To accomplish this, 500 µl of TMAH solution (pH ∼11) was added to 50 ml of deionized water containing 500 mg of raw Ni-X NP. The suspension was subjected to ultrasonication briefly, followed by stirring at room temperature for over 24 h. The hydroxylated Ni-X NP were centrifuged and washed 3 times with deionized water and 3 times with ethanol, then dried overnight at 70 °C and collected.

For –COOH modifications, 100 mg of the hydroxyl-modified Ni-X NP samples were each weighed into a flask containing 20 ml of toluene, followed by the addition of 3 ml of triethoxysilylpropyl succinic anhydride silane (TESPSA). The resulting mixture was refluxed at 110 °C for 24 h under an argon atmosphere to introduce the –COOH functional groups onto the Ni-X NP surface. The –COOH products were washed and oven-dried overnight (70 °C).

For surface modification with the –CH_3_ group, 100 mg of the hydroxyl-modified Ni-X NP sample was weighed into a flask containing 20 ml of toluene and fully sonicated for several minutes followed by the addtion of 3 ml of BTMS. The mixture was then refluxed at 110 °C for 24 h under an argon atmosphere to introduce –CH_3_ functional groups onto the Ni-X NP surface. After the reaction, the products were subjected to the same washing and drying procedures described above.

### Mice

Male and female C57BL/6 mice were used in this study. All mice were maintained in pathogen-free conditions (22 ± 2 °C, 30% to 40% humidity, 12 h light/12 h dark cycles) and offered food and water ad libitum in the animal facilities at the University of Montana (UM, Missoula, MT). Mice were sacrificed at 8 to 10 weeks of age. All experiments met the approval of the Institutional Animal Care and Use Committee (IACUC) of the University of Montana.

### Lung lavage

For mouse lung lavage, mice were euthanized by sodium pentobarbital, after which the lungs with the heart were removed, and the lungs were then lavaged. Briefly, 1 ml of ice-cold sterile phosphate-buffered saline (PBS) was instilled and withdrawn through a tracheotomy tube and repeated multiple times until a 5 ml volume of PBS had been retrieved. The fluid was then centrifuged at 400 × *g* for 5 min at 4 °C, the supernatant was aspirated and discarded, and the cell pellet was used for the cell count. Lung lavage cells were resuspended in 1 ml RPMI 1640 complete media (10% fetal bovine serum), counted, and adjusted to 10^6^ cells/ml.

### Ni-X NP stock particle suspensions

All Ni-X NPs, except for the methylated (–CH_3_) Ni-X NP variants, were weighed and suspended in freshly constituted PBS at 5 mg/ml. The Ni-X NP (–CH_3_) was weighed and suspended in DMSO at 20 mg/ml (note: Hydrophobicity of the methylated surface eliminated the option of initial suspension in aqueous buffer). This suspension was then diluted into PBS to create a 5 mg/ml stock solution and added into 100 μl medium over 1×10^5^ cells at a final particle concentration of 12.5 to 100 μg/ml and with DMSO ranging from 0.0625% to 0.5% in cell medium. The particle suspensions were then sonicated in a Masonix (Farmingdale, NY) cup-horn sonicator (XL2020, 40% power, 8000 Joules, 20 kHz, 550 watts) for 4 min.

### Cell cultures

AMs were suspended in RPMI 1640 complete media (Gibco, 11875119) supplemented with 10% fetal bovine serum, 0.01% 2-mercaptoethanol, 1% sodium pyruvate, and supplemented with 1% antimycotic/antibiotic cocktail (Mediatech, Manassas, VA). Cells were suspended at 1 × 10^6^ cells per ml, and then lipopolysaccharide (LPS, Sigma, St Louis, MO) at 20 ng/ml was added to stimulate pro-IL-1β formation via NF-kB activation. A 100 μl sample (1 × 10^5^ cells) of cells was exposed to each dose of Ni-X NP (e.g.: high dose 100 μg/ml, equivalent to 10 μg/10^5^ cells or 31.24 μg/cm^2^ [as calculated by 10 μg on 0.32 cm^2^ surface area of a 96-well plate]), and experiments were conducted in 96-well plates for 24 h in 37 °C water-jacketed CO_2_ incubators (ThermoForma, Houston, TX). Particle concentrations were 0, 12.5, 25, 50, and 100 μg/ml. Media was collected for the IL-1β assay, and cell viability was determined by lactate dehydrogenase (LDH) and MTS assays. Ten to 12 mouse lung lavage collections (described above) were pooled, and this experiment was replicated at least 3 times.

### Quantification of Ni-X NP uptake for AMs

Flow cytometric side scatter was used to quantify cell/particle binding and uptake in a similar manner as previously described (Hamilton Jr et al. 2006). In this study, following Ni-X NP exposure for 1 h, AM were pelleted, washed twice with PBS, and resuspended in 0.5 ml of PBS, then analyzed immediately on an Attune NxT flow cytometer using Attune software (BD Biosciences). Flow cytometric methods detected Ni-X NP uptake into the AM by assaying for changes inside scatter properties while gating on AM. Data are expressed as average median side scatter intensity with arbitrary numerical units.

### Toxicity assays

Cell viability was determined by both MTS using the CellTiter^96^ assay (Promega, Madison, WI) and LDH detection using the CytoTox^96^ assays. These assays used a colorimetric dye read by a colorimetric plate reader (Molecular Devices, Sunnyvale, CA) at 490 nm. The LDH assay was performed according to the manufacturer’s instructions (Promega). However, for the MTS assay, an extra step was taken to remove the developed MTS reagent (transferring it into another plate) from the cell/particle mixture adhered to the plate bottom to avoid particle-caused artifacts in the optical density values. Data for the LDH assay is expressed as % LDH release compared with 100% cell lysis. Data for the MTS assay are relative to the no-particle control culture optical density values.

### Cytokine assays

An ELISA assay was performed using a mouse IL-1β DuoSet ELISA kit (R&D Systems) according to the manufacturer’s protocol. ELISA was performed on supernatants from cells plated with LPS and Ni-X NPfor 24 h. Plates were read at 450 nm, and data expressed as pg/ml as determined by standard curve.

### RBC hemolysis assay

The RBC hemolysis assay was used to determine the potential of Ni-X NP to cause lysosomal membrane permeability (LMP) in a similar manner as previously described (Sydor et al. 2021; Pavan et al. 2022). Human RBCs obtained from Innovative Research (Novi, MI) were diluted 1:10 in PBS, and 1 ml was placed into 1.5 ml tubes. The stock Ni-X NP suspensions were prepared at 20 mg/ml in PBS or DMSO and added at 200, 400, or 600 µg/ml to RBC suspensions. DMSO vehicle controls (1%, 2%, and 3%) were run on RBC with no apparent effect (results not shown). The RBC–Ni-X NP suspensions were mixed by gentle inversion and placed in a rotating tube holder (LabQuaker) at 37 °C for 4 h. At the end of that period, the samples were centrifuged for 10 min at 5000 RPM at room temperature; 100 µl of the supernatant was pipetted into a 96-well plate in triplicate. Drabkin’s reagent (Sigma, St Louis, MO) was added at 100 µl/well, and the plate was incubated for 15 min in a plate shaker (low speed) at room temperature. The absorbance was read at 540 nm. The data are expressed relative to 100% RBC lysis established by adding 10% triton X 100 at the beginning of the incubation. RBC hemolysis data are presented as fold increase over the individual’s no particle control, which varied from 0% to 12%, to account for the large variance in human RBC membrane response.

### Determination of Ni^2+^ released from Ni-X NP

To quantify Ni^2+^ ion release from the different Ni-X NPs, particles suspended in PBS at known concentrations (mg/ml) were added to 250 μl of SLF. The SLF was prepared as described by Yousef et al. (2023). That is, NaCl, KCl, CaCl2•2H2O, sodium acetate, and acetic acid were dissolved in deionized water, then pH was adjusted to 4.5 with HCl. To simulate Ni-X NP interactions with lysosomal contents, nanoparticles were incubated with tumbling (37 °C) for 1 h in SLF. Particles were pelleted by centrifugation (10,000 RPM, 5 min) and supernatants removed immediately. Ni^2+^ in supernatants was assessed by treating with dimethylglyoxime (DMG; 4 μM) and quantifying absorbance at 543 nm after 5 min. DMG forms a bright pink insoluble product when chelating Ni^2+^ ions, allowing Ni^2+^ to be quantified by measuring optical density. Ni^2+^ concentration was further evaluated by comparing optical density at 543 nm to a standard curve of known Ni^2+^ concentrations using a dissolved nickel salt (NiCl).

### The murine ex vivo AM model

The mexAM cells were generated as previously described (Gorki et al. 2022; Albright et al. 2023; Kendall et al. 2023). Briefly, primary AM were isolated by whole lung lavage from C57BL/6 mice, aged 8 to 12 weeks, as described above. Cells were cultured in RPMI 1640 complete media (10% FBS, 1% Penicillin/Streptomycin/Amphotericin B, 1% Sodium Pyruvate, 0.1% 2-mercaptoethanol) with murine GM-CSF (30 ng/ml), recombinant hTGFβ (10 ng/ml), and PPARγ agonist rosiglitazone (1 µM). Media and growth factors were replaced every 5 days, and cells were passaged at 80% to 90% confluency (∼5 to 7 days). Cells were collected using lidocaine and gentle scraping and used in the described experiments. All experiments were performed between passages 5 and 15.

### Determination of LMP

LMP was assessed by measuring the activity of the lysosomal enzyme Cathepsin B in cytosol extracts from mexAM exposed to Ni-X NP. Briefly, cells were incubated with 50 μg/ml of NCZ, SIG, and AA in black/clear-bottom 96-well plates at 37 °C with 5% CO_2_ for 4 h, a time previously optimized for the detection of LMP by nanoparticles (Jessop et al. 2017). Following Ni-X NP exposure, the cells were washed twice with PBS and then incubated on ice with 100 μl/well of digitonin reaction buffer for 5 min with gentle rocking. The digitonin reaction buffer (pH 7.5) contained sucrose (250 mM sucrose, Sigma Aldrich, 57-50-1), HEPES (20 mM, Sigma Aldrich, 7365-45-9), KCl (10 mM, Sigma Aldrich, P-3911), MgCl_2_ (1.5 mM, Sigma Aldrich, M8266), EDTA (1 mM, Thermo Fisher, AM9260G), EGTA (1 mM, Fluka, 03779), pefabloc (0.5 mM, Sigma Aldrich, 76307), and digitonin (20 μg/ml, EMD Millipore, 300410). The digitonin concentration was previously optimized by titration (data not shown) to maximize plasma membrane permeability (indicated by LDH release into supernatant) while simultaneously minimizing lysosome membrane disruption (indicated by cathepsin B activity in supernatant). Cathepsin B activity was measured by reaction of 40 μl of cytosol extracts with 40 μl of the fluorogenic substrate Z-Arg-Arg-7-AMC (50 μM, Echelon, 88937-16-5) in cathepsin B reaction buffer (pH 6), which contained sodium acetate (50 mM, Sigma Aldrich, 127-09-3), EDTA (4 mM, VWR, 6381-92-6), pefabloc (0.5 mM, Sigma Aldrich, 76307), and dithiothreitol (8 mM, VWR, 0281). The samples were assessed on a plate reader (Molecular Devices, model SpectraMax iD3) using a 20-min kinetic read with 45-s intervals at 30 °C and ex/em 365/440. V_max_ for the liberation of 7-amino-4-methylcoumarin (AMC) from each well was normalized to the optical density of the cytosolic protein LDH for the same well (CytoTox 96, described above). The adjusted values were plotted as a fold-change against negative control cells.

### Statistical analyses

Statistical analyses involved comparison of means using a one-, two-, or three-way *ANOVA* followed by Dunnett’s test or Holm–Sidak’s adjustment to compensate for increased type I error resulting from pair-wise mean comparisons. All probabilities were two-tailed unless otherwise stated. Statistical power was greater than 0.8. Statistical significance was defined as a probability of type I error occurring at less than 5% (*P *< 0.05). The minimum number of sample replications was 4, and the maximum was 8. Graphics and analyses were performed on PRISM v.7.0 (GraphPad, San Diego, CA).

## Results

### Component and structure of Ni-X NP

**Fig. 1.**
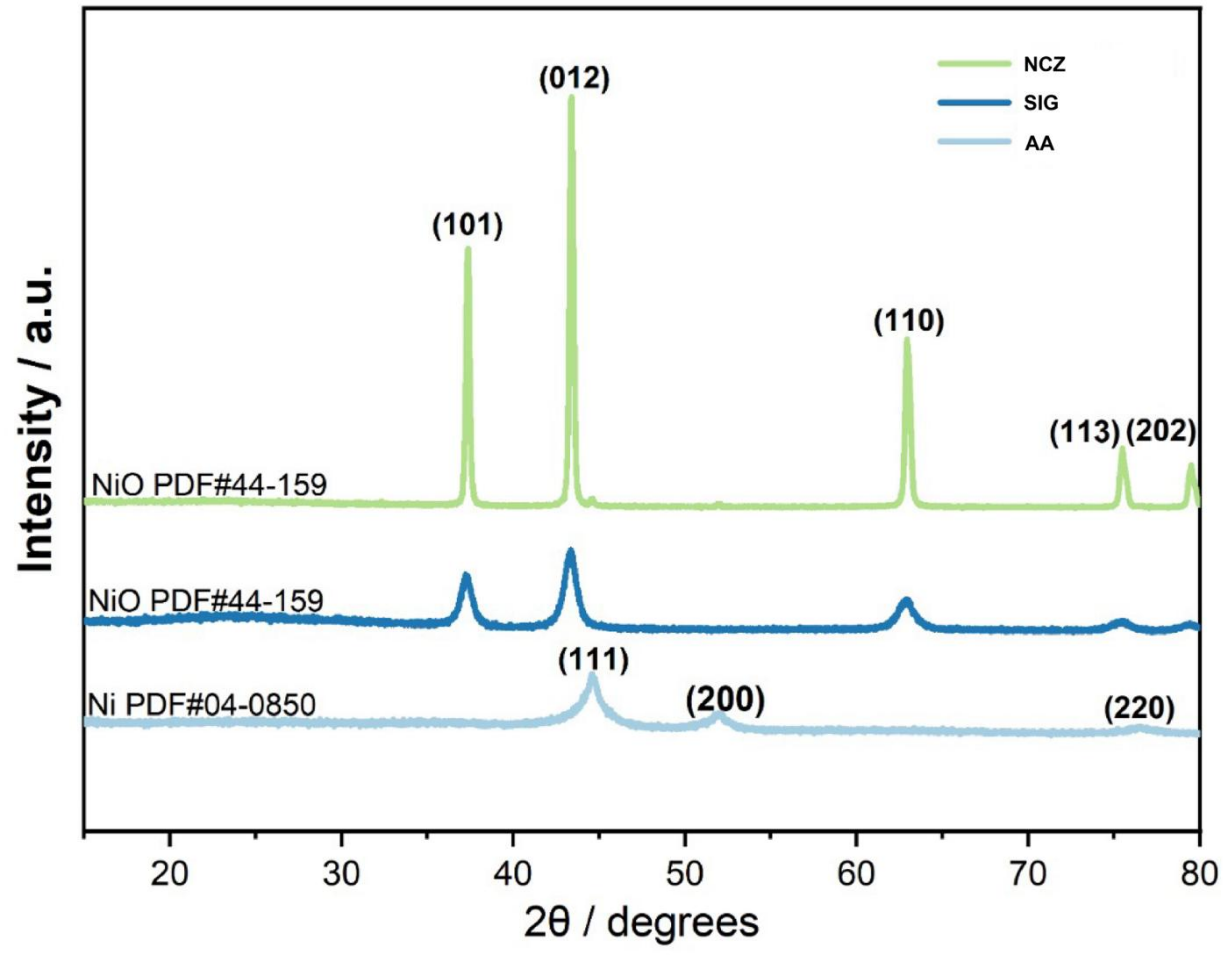
XRD pattern diffraction spectra for Ni-X NP.

Three Ni-X NPs used in this study are denoted as: (i) NCZ, (ii) SIG, and (iii) AA. Powder XRD was used to analyze the bulk crystal structure of the 3 raw samples. As shown in Fig. 1, the observed diffraction peaks of AA at 2θ = 44.643°, 52.041°, and 76.564° were assigned to the (111), (200), and (220) crystal planes of bulk Ni. In contrast, the primary diffraction peaks of SIG and NCZ at 2θ = 37.307°, 43.334°, 62.874°, 75.513°, and 79.555° corresponded to the (101), (012), (110), (113), and (202) crystal planes of bulk NiO. Additionally, the higher peak intensity and narrower peak width of NCZ indicated a higher degree of crystallinity than either SIG or AA. Crystallinity of Ni-X NP followed the trend NCZ ≫ SIG > AA.

**Fig. 2.**
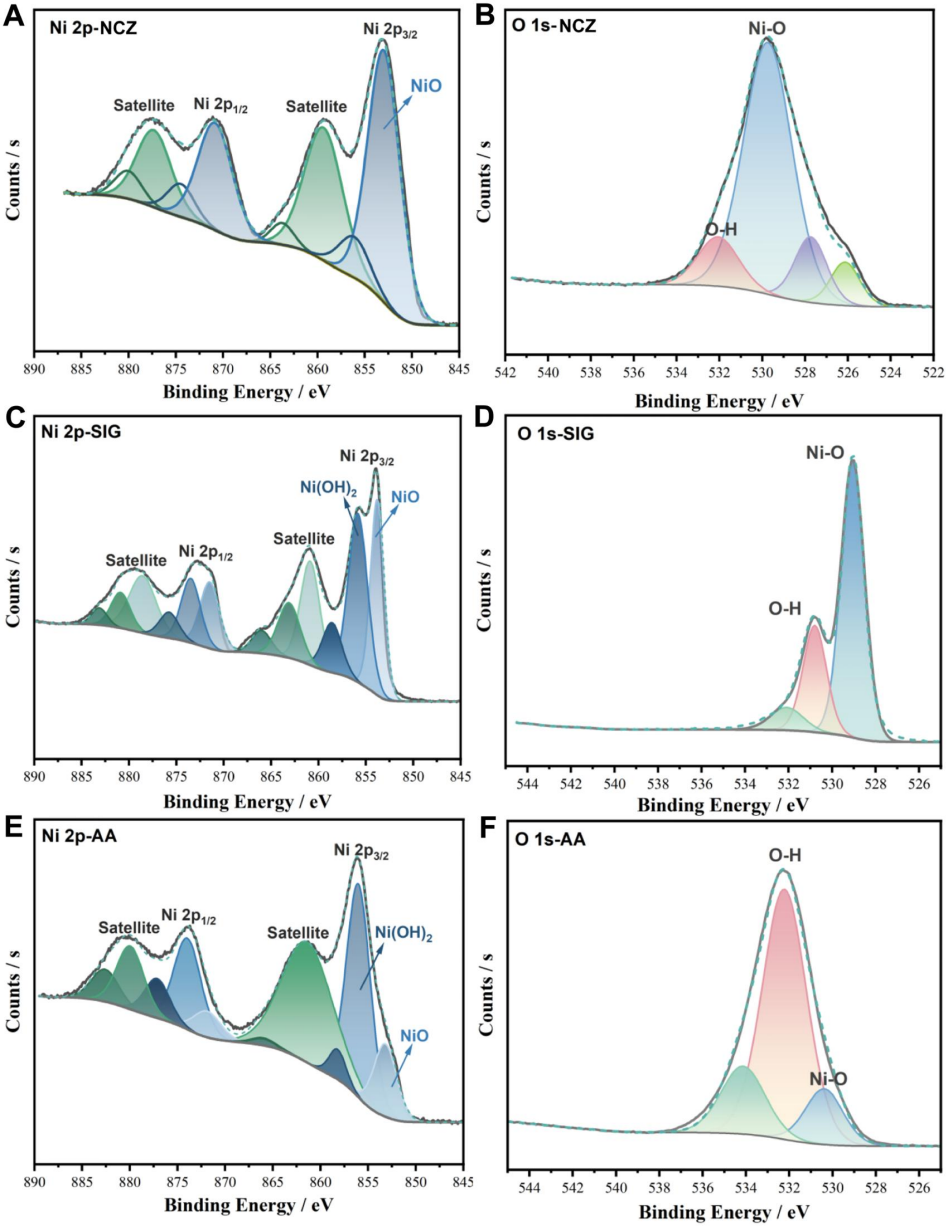
The Ni 2p and O 1s XPS peaks of raw Ni-X NP. A and B) NCZ, C and D) SIG, and E and F) AA.

**Fig. 3.**
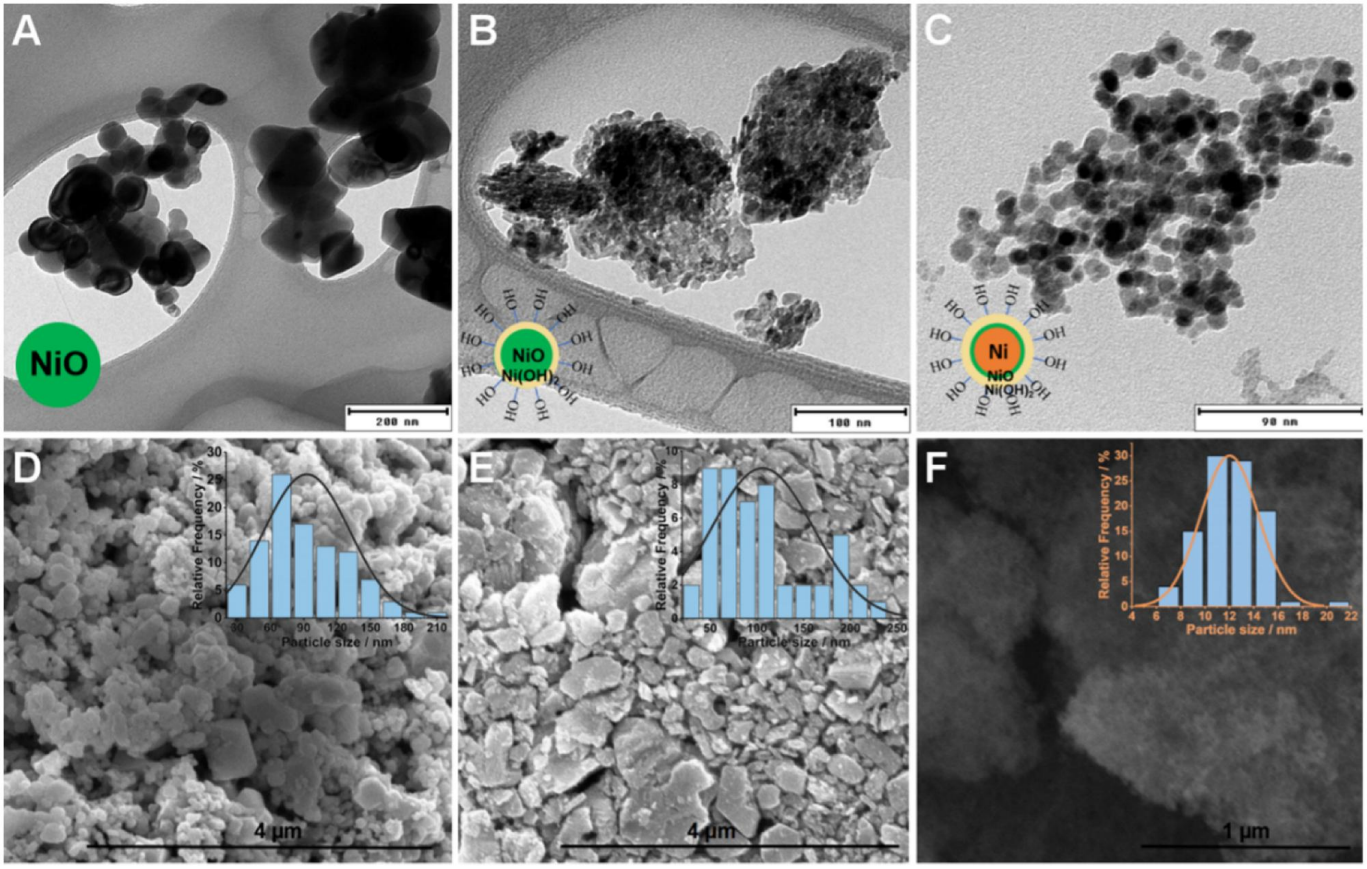
Electron microscopy images and size distributions of Ni-X NP. TEM images and corresponding size distribution of A) NCZ (pure NiO), B) SIG (NiO-core@Ni(OH)_2_-shell), and C) AA (Ni-core@NiO@Ni(OH)_2_-shell). SEM images of D) NCZ, E) SIG, and F) AA.

XPS was used to analyze the surface chemical states and elemental composition of the 3 samples. According to the Gaussian–Lorentzian function, the Ni 2*p* spectrum of NCZ (Fig. 2A) displayed 2 main peaks at 853.1 and 870.9 eV, confirming NiO as the predominant surface component. The O 1 *s* spectrum (Fig. 2B) further supported this, with peaks at 529.7 and 532.1 eV attributed to lattice oxygen and chemisorbed –OH, respectively. For SIG, in addition to the characteristic peaks of NiO at 853.8 and 871.5 eV, the presence of additional peaks at 855.9 and 873.5 eV confirmed the existence of Ni(OH)_2_ (Fig. 2C). The O 1 *s* spectrum (Fig. 2D) showed a strong surface hydroxyl signal at 530.8 eV. AA’s Ni 2p spectrum (Fig. 2D) identified Ni(OH)_2_ as the dominant surface component, while NiO was still present. The O 1 *s* spectrum (Fig. 3F) further confirmed surface hydroxyl groups with a peak at 532.2 eV. Taken together, the results of the XRD and XPS analyses suggest that NCZ was pure NiO, whereas SIG followed a bilayer NiO@Ni(OH)_2_ structure, and AA exhibited a tri-layer Ni@NiO@Ni(OH)_2_ configuration (illustrated in the insets in Fig. 3).

### Morphology characterization of Ni-X NP

The morphology of the 3 raw Ni-X NPs was characterized by using TEM and scanning electron microscopy (SEM). This characterization revealed significant differences in particle morphology and size among the 3 samples. NCZ exhibited a broad particle size distribution with an average size of 90 nm (Fig. 3A and D). SIG showed a distinct disordered mesoporous structure with an average size of 110 nm (Fig. 3B and E). These mesoporous walls were comprised of small, highly crystalline nanocrystals with an individual size of 10 nm. AA showed a more concentrated distribution around 12 nm (Fig. 3C and F). Overall, the Ni-X NP demonstrated a size range of SIG > NCZ ≫ AA.

### Surface modification of Ni-X NP

**Fig. 4.**
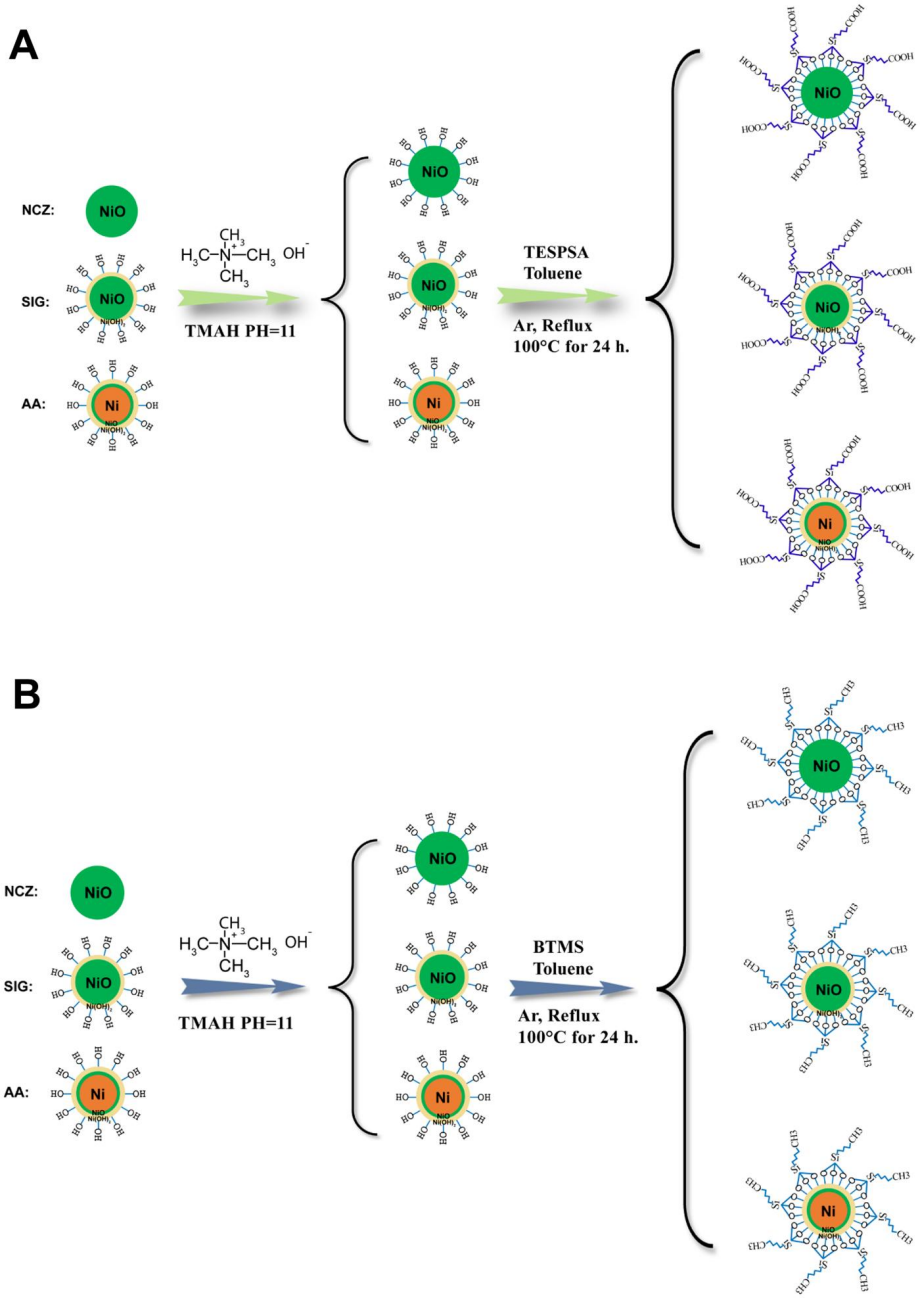
Schematic illustration of A) the surface –COOH functional group modification of the raw Ni-X NP or B) the surface –CH_3_ functional group modification of the raw Ni-X NP.

**Fig. 5.**
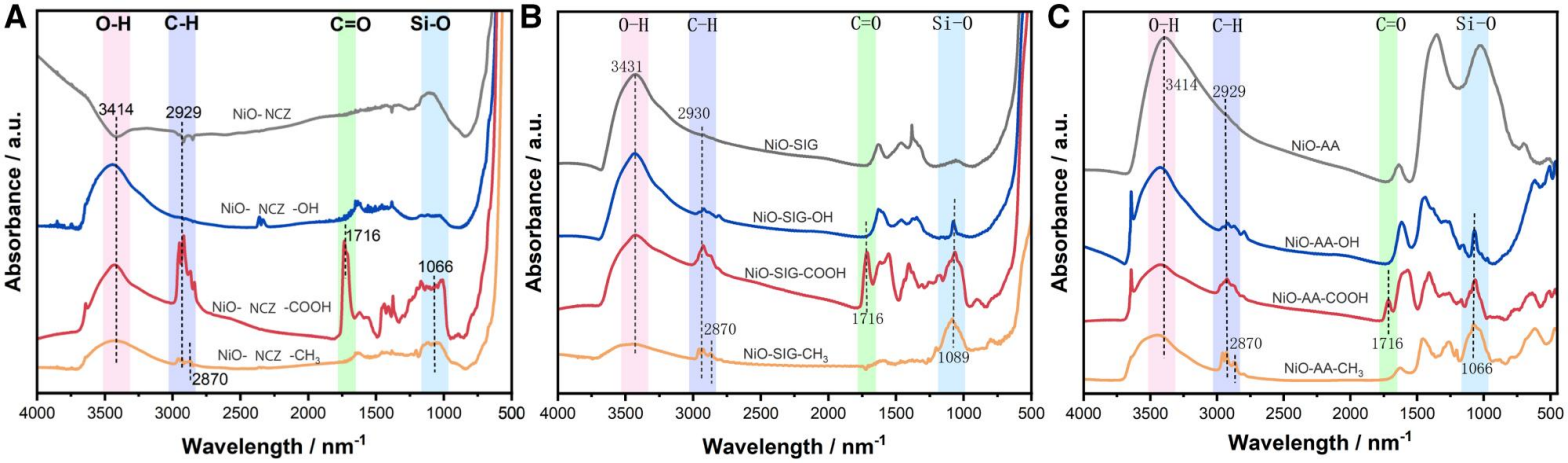
FTIR characterization of different functional group on A) NCZ, B) SIG, or C) AA.

Three different raw NCZ, SIG, or AA Ni-X NP possess surface metal oxide layers, which are partially hydrolyzed in the alkaline environment provided by TMAH to generate surface metal hydroxyl groups. This process introduced abundant –OH groups onto the nanoparticle surfaces, creating effective active sites for subsequent silane coupling reaction. The silanol groups (Si–OH) generated by hydrolysis of TESPSA can undergo condensation reactions with the surface –OH groups, covalently anchoring the –COOH functional groups onto the nanoparticles (Fig. 4A). Similarly, BTMS also undergoes hydrolysis followed by condensation with surface –OH groups, resulting in the surface –OH being capped with hydrophobic –CH_3_ groups (Fig. 4B). Fourier transform infrared spectrum (FTIR) was used to characterize the –OH, –COOH, and –CH_3_ modifications on the raw NCZ (Fig. 5A), SIG (Fig. 5B), and AA (Fig. 5C). The broad absorption peak observed at 3500 cm^−1^ was attributed to O–H stretching vibrations. Following the introduction of –COOH groups, a distinct C = O stretching vibration peak appeared near 1700 cm^−1^. Upon –CH_3_ functionalization, symmetric and asymmetric C–H stretching vibrations were identified at 2930 and 2870 cm^−1^, respectively. Additionally, the characteristic Si-O vibration peaks detected in the 1000 to 1100 cm^−1^ range confirmed the formation of Si–O–Si bonds during TESPSA and BTMS modifications.

### Zeta potentials of raw and modified Ni-X NP

ENM surface charge is known to play a role in nanoparticle agglomeration of the particle as well as in interactions of nanoparticles with cell membranes (Bonner and Brown 2020). Nanoparticles can exhibit a change of zeta potential in the presence of protein coronas; as such, it was important to consider the dispersion method of nanoparticles in a cellular model. Here, surface charge distribution of the raw and modified samples was analyzed in the same complete RPMI 1640 media that would be used in nanoparticle exposures in macrophages. As shown in Table 1, all 3 raw samples exhibited negatively charged surfaces, with the average zeta potential following the order NCZ > AA > SIG. Functionalization of NCZ with –OH or –COOH caused the zeta potential to increase, whereas no significant change was observed for the –CH_3_-modified sample. Functionalization of SIG with –OH or –COOH caused a decrease in the zeta potential, as did –CH_3_ to a lesser degree. When AA was functionalized, there was no change in the zeta potential with –OH or –COOH, but an increase in the zeta potential was observed with –CH_3_. Taken together, there is no demonstratable pattern of change in the zeta potentials by surface modification of NCZ, SIG, and AA.

**Table 1.** Zeta potential summary of Ni-X NP in complete media.

Average zeta potential (mV)	NCZ	SIG	AA
**Raw**	−9.47	−7.22	−8.71
**–OH**	−7.49	−11.32	−8.35
**–COOH**	−5.68	−10.70	−8.03
**–CH_3_**	−9.26	−8.21	−6.297

### Analysis of Ni-X NP hemolytic capability

**Fig. 6.**
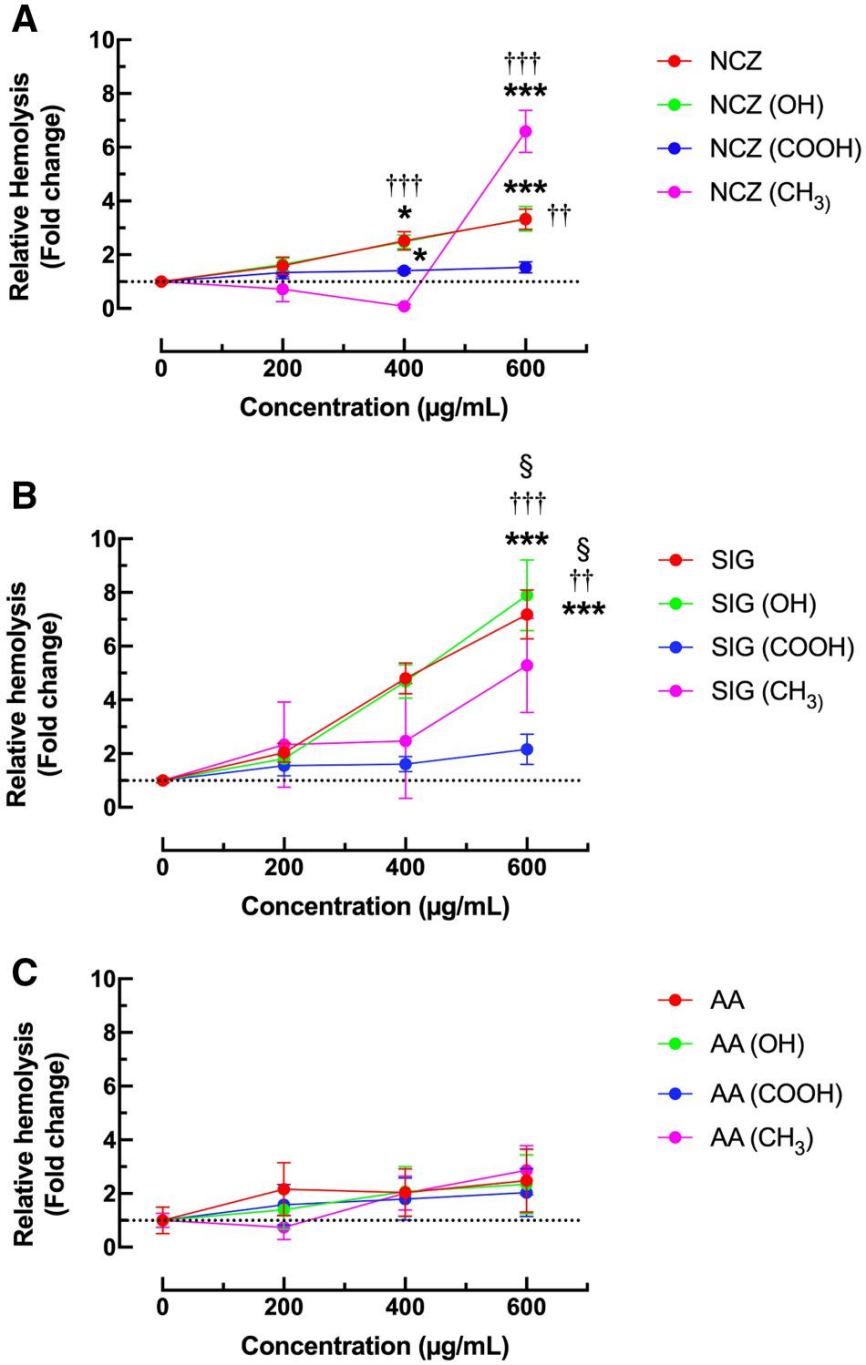
Hemolysis of human RBC at 4 h exposure to Ni-X NP. A) NCZ, B) SIG, and C) AA. Data expressed as mean ± SEM fold increase normalized to 0 μg/ml. Asterisks indicate significance compared to 0 μg/ml. Daggers indicate significance compared to the other Ni-X NP at the same dose. Section symbol indicates significance compared to other Ni-X NP with same functionalization. One symbol: *P* < 0.05, 2 symbols: *P* < 0.01, and 3 symbols: *P* < 0.001. *n* = 6 experimental replicates.

A potential mechanism of particle-induced IL-1β production in AM is LMP. This can be modeled by examining hemolysis in human RBCs incubated with the particles for 4 h, tumbling at 37 °C. RBCs are not phagocytic; therefore, the nanoparticles are not taken up by RBC. Yet, surface interactions between the particle and RBC can disrupt the membranes and result in the release of hemoglobin. Because of the lack of phagocytic uptake, the concentration of particles necessary for this particular assay is generally higher than that for cytotoxicity assays in macrophages. NCZ and NCZ–OH had identical effects, increasing hemolysis in a dose-dependent style, resulting in a 3-fold increase in hemolysis at the highest concentration (Fig. 6A). NCZ–COOH failed to produce hemolysis and was statistically different from the other Ni-X NP effects. NCZ–CH_3_ showed a significant increase in hemolysis only at the highest concentration. SIG variants produced a very similar pattern to NCZ (Fig. 6B). Raw and –OH functionalized produced similar results in a dose-dependent manner and essentially doubled the hemolysis levels over NCZ to produce an overall 7-fold increase. SIG–COOH had no effect on the RBC membranes at any dose. SIG–CH_3_ produced a moderate increase in hemolysis that was not statistically significant even at the highest dose. In contrast, AA and all functionalizations failed to produce significant hemolysis at any nanoparticle concentration (Fig. 6C).

### Quantitation of Ni^2+^ release in artificial lysosome fluid for the Ni-X NP

**Fig. 7.**
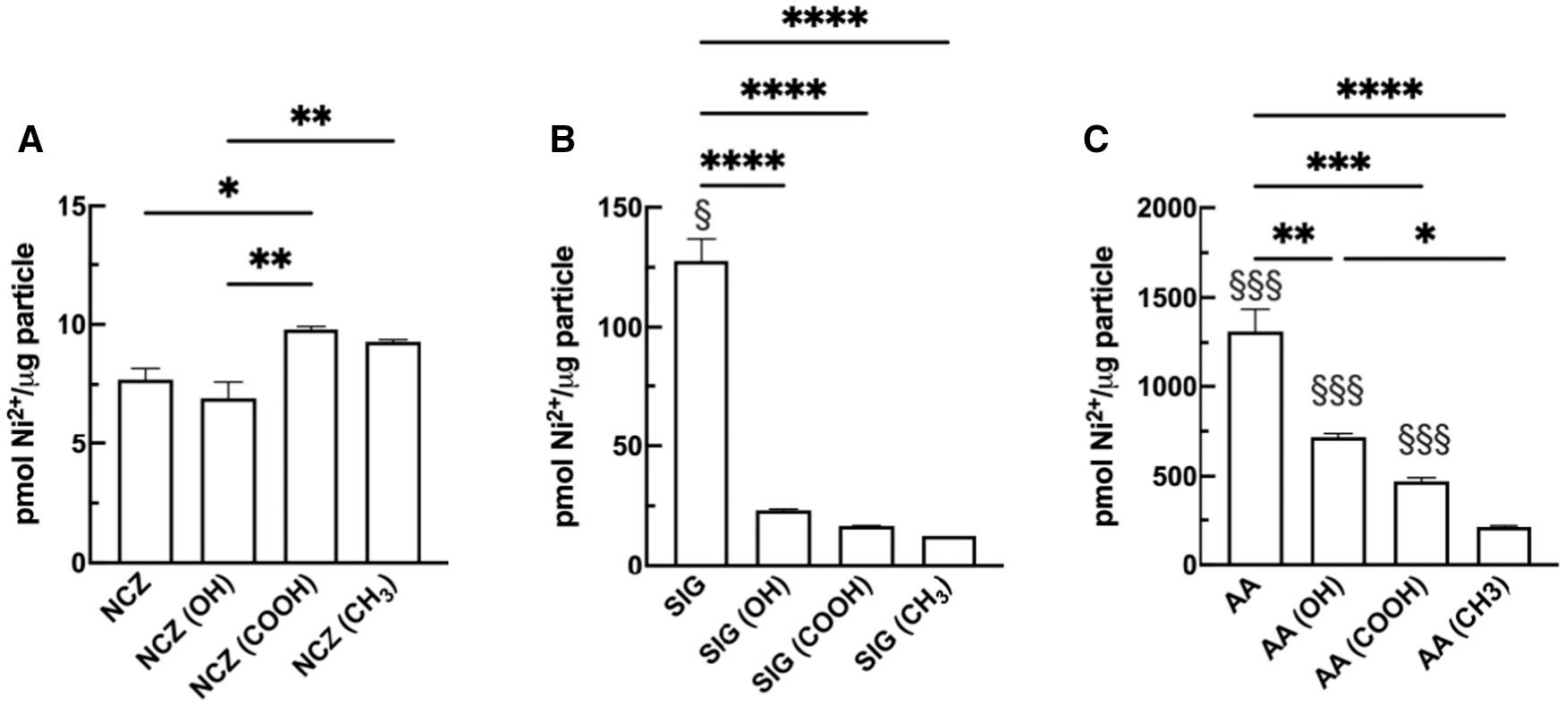
Ni^2+^ ion release by A) NCZ, B) SIG, and C) AA nanoparticles incubated in artificial lysosomal fluid for 1 h. Data expressed as mean ± SEM pmol Ni^2+^/μg Ni-X NP. Asterisks indicate significance compared to respective raw Ni-X NP. Section symbols indicate significance to other Ni-X NP of same functionalization. One symbol: *P* < 0.05, 2 symbols: *P* < 0.01, 3 symbols: *P* < 0.001, 4 symbols: *P* < 0.0001. *n* = 4 experimental replicates.

Ni^2+^ are reported to produce cell death and IL-1β release at sufficiently high doses (Ferko and Catelas 2018; Guo et al. 2019). Here, the role of Ni^2+^ released by internalized nanoparticles inside macrophages was considered as a possible mechanism of toxicity/inflammation. The acidic environment of the phagolysosome could be causing the Ni^2+^ to dissociate from the particle and become a soluble agent targeting mitochondria. NCZ, SIG, and AA nanoparticles were incubated in artificial lysosomal fluid and Ni^2+^ release was quantified. NCZ and its functionalized forms showed the least amount of Ni^2+^ release compared with the other nanoparticles (Fig. 7A). SIG had 10× the Ni^2+^ compared with NCZ (Fig. 7B), and AA nanoparticles demonstrated the greatest Ni^2+^ release (Fig. 7C). All functionalizations of SIG and AA showed a reduction in Ni^2+^, whereas NCZ showed a subtle increase in Ni^2+^ release with –COOH functionalization. The data trends were AA ⋙ SIG > NCZ. Within the AA and SIG moieties the trends were raw Ni-X NP > Ni-X NP(–OH) > Ni-X NP(–COOH) > Ni-X NP (–CH_3_).

### Evidence for the uptake of Ni-X NP

**Fig. 8.**
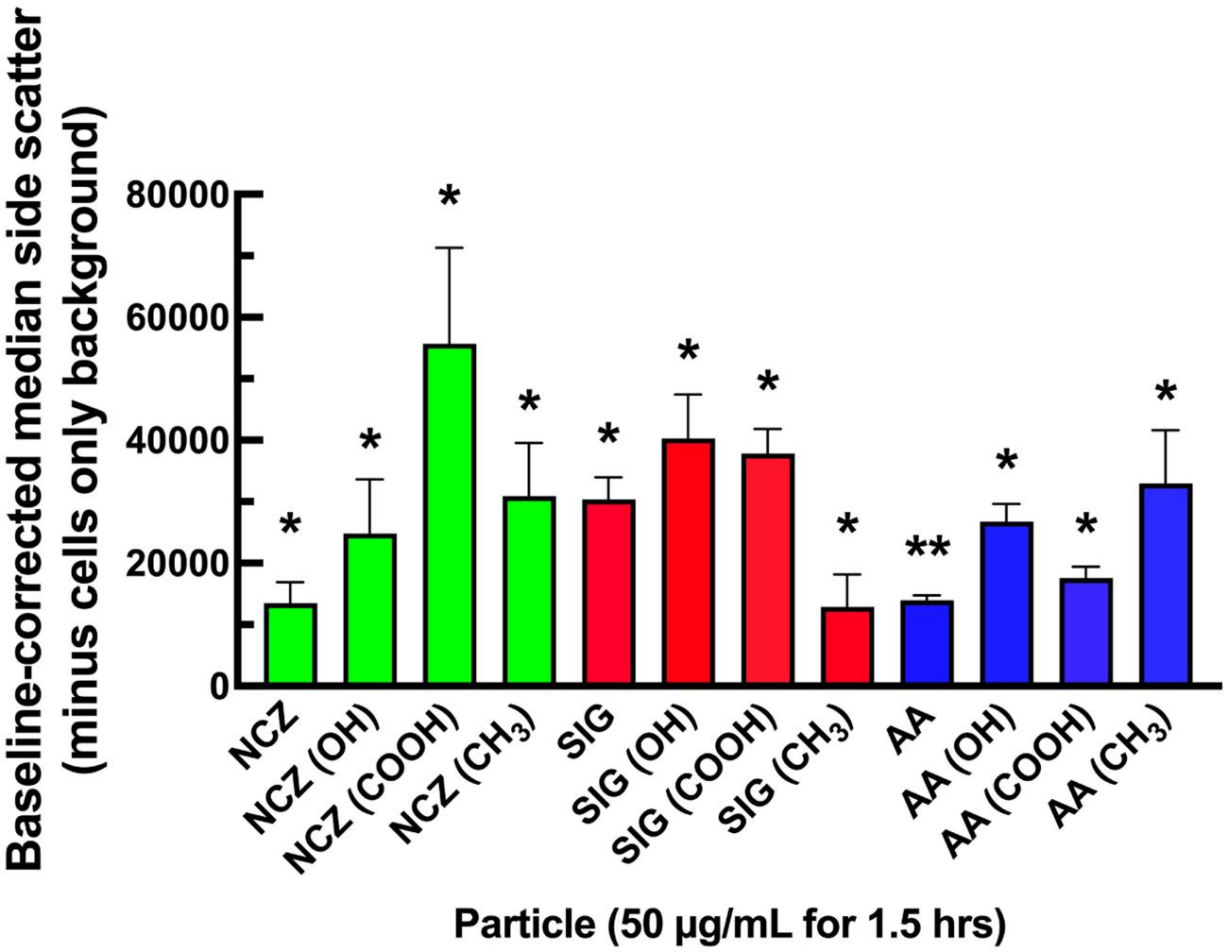
Ni-X NP particle uptake by alveolar macrophages. Median side scatter values of AM treated with Ni-X NP for 1 h were assessed by flow cytometry. Data expressed as mean ± SEM median side scatter, with the baseline side scatter (cells only) subtracted from treatment groups. * Indicates *P* < 0.05 and **P < 0.01 compared to untreated cells. Green: NCZ Ni-X NP; Red: SIG Ni-X NP; Blue: AA Ni-X NP. *n* = 3 experimental replicates.

After characterization and functionalization, particle uptake of Ni-X NP by murine AM was confirmed. Particle uptake was determined by evaluating changes to the median side scatter of cells following incubation with 50 μg/ml of the various Ni-X NPs for 1 h. Previously, changes in median side-scatter (SSC) compared with control have been described to indicate uptake of particles by phagocytic cells (Suzuki et al. 2007; Zucker and Boyes 2020). AM showed differing increases in median side scatter, indicating that nanoparticles had been taken up by AM (Fig. 8). AM showed a similar increase in median side scatter for NCZ and AA, whereas SIG showed a more marked increase. The inherent differences in light-scattering characteristics of different Ni-X NPs and functional groups make direct comparisons of particle uptake challenging. For example, others have reported an increase in light scattering of nanoparticles functionalized with (–COOH) (Mutzke et al. 2015). Thus, although there was significant variability in median SSC for each particle and functionalization, no comparison was made apart from a comparison to control cells. All Ni-X NP and functionalizations differed significantly from untreated AM. The binomial probability of all 12 particles increasing SSC by chance was less than 0.001.

### Cell death in AM following Ni-X NP exposure

**Fig. 9.**
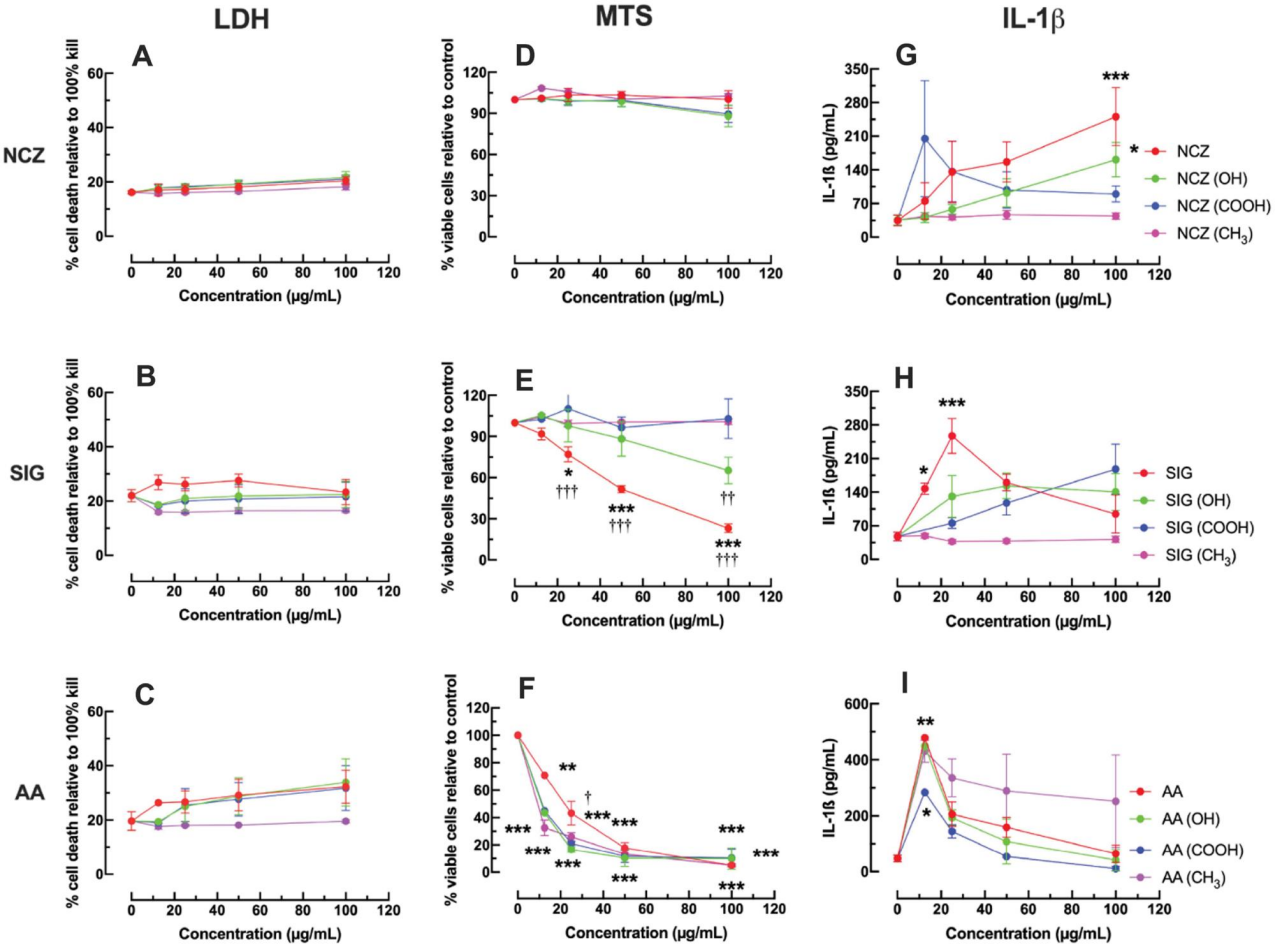
Cytotoxicity, cell viability, and IL-1β release of primary AM exposed to NCZ, SIG, and AA Ni-X NP. LPS-primed (20 ng/ml) AM were exposed to Ni-X NP for 24 h and cell death analyzed by release of lactate dehydrogenase (LDH) in cell supernatants. Percent cell death relative to 100% cell lysis control for A) NCZ, NCZ (–OH), NCZ (–COOH), and NCZ (–CH_3_); B) SIG, SIG (–OH), SIG (–COOH), and SIG (–CH_3_); and C) AA. AA (–OH), AA (–COOH), and AA (–CH_3_). Data expressed as mean ± SEM percent relative to total cell lysis. Cell viability was measured by mitochondrial metabolism by using MTS assay. D) NCZ, NCZ (–OH), NCZ (–COOH), and NCZ (–CH_3_). E) SIG, SIG (–OH), SIG (–COOH), and SIG (–CH_3_). F) AA, AA (OH), AA (COOH), and AA (–CH_3_). Data expressed as mean ± SEM percent color development (optical densities converted to percent) relative to the no particle (0 μg/ml) culture control (100% viability at 24 h). IL-1β release (proxy metric for LMP) was assessed at 24 h. G) NCZ, NCZ (–OH), NCZ (–COOH), and NCZ (–CH_3_). H) SIG, SIG (–OH), SIG (–COOH), and SIG (–CH_3_). I) AA, AA (–OH), AA (–COOH), and AA (–CH_3_). Data expressed as mean ± SEM IL-1β in pg/ml. Asterisks indicate significance compared to 0 μg/ml particle concentration. Daggers indicate significance compared to the raw Ni-X NP at the same dose. One symbol: *P* < 0.05, 2 symbols: *P* < 0.01, and 3 symbols: *P* < 0.001. *n* = 3 to 9 experimental replicates.

AM were cultured with NCZ, SIG, and AA Ni-X NP (12.5, 25, 50, and 100 μg/ml) and LPS (20 ng/ml) for 24 h. After 24 h, cell death was evaluated by release of LDH into the supernatants of treated cells. None of the Ni-X NPs tested caused a significant increase in cell death at the 24 h time point. NCZ produced a dose–response curve with the smallest slope, showing negligible cell death for all variations and for all doses (Fig. 9A). SIG-treated AM demonstrated slightly more cell death as the raw SIG particle caused an increase in cell death at 12.5, 25, and 50 μg/ml that trailed off at 100 μg/ml (Fig. 9B). Additionally, SIG (–OH), SIG (–COOH), and SIG (–CH_3_) appeared to reduce cell death compared with raw SIG, though not to significance (Fig. 9B). The AA Ni-X NP showed a subtle dose–response for AA, AA (–OH), and AA (–COOH), but no cell death for any dose of AA (–CH_3_) (Fig. 9C). However, it should be noted that none of the cell death values were statistically significant when compared with control cells, suggesting an overall lack of cell membrane permeabilization for the panel of Ni-X NPs.

### Cell viability in AM following Ni-X NP exposure

Cell viability was assessed in AM treated with Ni-X NP. In contrast to the LDH assay, which showed no demonstratable cell death, the MTS assay demonstrated a reduction in cell viability due to particle exposure in AM. The MTS assay relies on the NADPH and NADH produced by dehydrogenase enzymes in metabolically active cells to create a colored product (Dunigan et al. 1995). NCZ nanoparticles had negligible impact on cell viability with no measurable difference in recorded optical density for (–OH), (–COOH), or (–CH_3_) functionalization (Fig. 9D). Raw SIG showed a significant reduction in cell viability in a dose-dependent manner that was alleviated with (–OH), (–COOH), and (–CH_3_) functionalization at doses of 25 μg/ml and above (Fig. 9E). Raw AA Ni-X NP caused a profound reduction in cell viability (Fig. 9F), even at the lowest dose (12.5 μg/ml). In this case, functionalization of AA nanoparticles failed to recover cell viability; rather, AA(–OH), AA(–COOH), and AA(–CH_3_) demonstrated even further reduced cell viability at 12.5 μg/ml (Fig. 9F). These results differ from the LDH results for SIG and AA particles. NCZ raw and functionalized particles showed a cell viability that corroborated the LDH results, suggesting a complete lack of bioactivity for the NCZ particles at the given doses for 24 h.

**Fig. 10.**
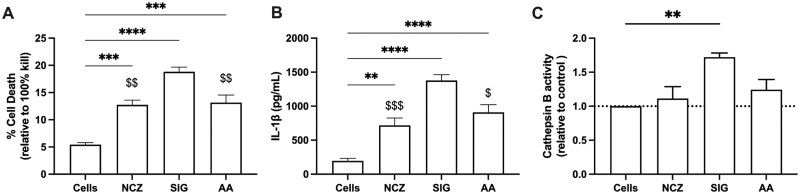
Ni-X NP-induced cytotoxicity, IL-1β, and LMP in mexAM macrophages. LPS-primed mexAM were cultured with NCZ, SIG, or AA for 24 h. A) Cell death as indicated by LDH release. B) IL-1β release in supernatants. Data expressed as mean ± SEM. *n* = 4. C) Cathepsin B measured in cytosolic extracts at 4 h in mexAM. Data are expressed as mean ± SEM fold change in cathepsin B activity compared to control cultures. *n* = 3 experimental replicates. Asterisks indicate significance compared to control cells. Dollar sign symbols indicate significance to SIG Ni-X NP. One symbol: *P* < 0.05, 2 symbols: *P* < 0.01, 3 symbols: *P* < 0.001, and 4 symbols: *P* < 0.0001.

### IL-1β release in Ni-X NP exposed AM

The inflammatory potential of Ni-X NP was evaluated in LPS-primed AM. AM were cultured with LPS (20 ng/ml) and nanoparticle (50 μg/ml) for 24 h, and IL-1β release was measured. Raw NC demonstrated a linear dose–response with a positive slope that reached statistical significance at 100 μg/ml (Fig. 9G). NCZ(–OH) had a smaller slope than raw NCZ but also reached statistical significance at 100 μg/ml. NCZ(–COOH) spiked to its highest output of IL-1β at 12.5 μg/ml and decreased at each dose thereafter. NCZ(–CH3) showed no change in IL-1β compared with control cells. Raw SIG demonstrated a dose–response curve that peaked at 25 μg/ml and declined thereafter (Fig. 9H); 12.5 and 25 μg/ml doses of SIG were statistically increased above untreated cells. SIG–OH showed a similar curve to raw SIG but was reduced in magnitude. SIG(–COOH) showed a linear dose–response curve. SIG(–CH3) produced no IL-1β. AA nanoparticles had a similarly shaped dose–response curve regardless of functionalization and displayed little variation in amplitude with functionalization vs raw (Fig. 9I). All AA demonstrated a significance in IL-1β release at 12.5 μg/ml.

### Evaluation of mexAM treated with raw Ni-X NP

Previously, mexAM were reported as a useful method for studying tissue-resident macrophage function and behavior (Gorki et al. 2022). We have reported that mexAM demonstrate a robust IL-1β response to nano- and micron-sized particles (Kendall et al. 2023). Use of this cell model is advantageous as it allows for the IL-1β response to move away from the baseline (noise) of the assay demonstrated in AM. In fact, mexAM were shown to be especially sensitive to raw SIG, showing a greater than 10-fold increase in IL-1β when compared with AM for all concentrations tested (Kendall et al. 2023). Here, mexAM were primed with LPS (20 ng/ml) as previously described (Hamilton et al. 2009) and treated with raw Ni-X NP (50 μg/ml) for 24 h. NCZ, SIG, and AA showed a moderate but significant increase in cell death compared with untreated cells (Fig. 10A). SIG caused significantly higher cell death than either NCZ or AA. IL-1β release from mexAM treated with Ni-X NP showed a significant increase over baseline for NCZ, SIG, and AA (Fig. 10B), with SIG having significantly higher IL-1β release compared with either NCZ or AA. LMP disrupts lysosomal membranes and causes the release of cathepsin B and other proteases from the lysosomal space (Heid et al. 2013; Katsnelson et al. 2016; Bunderson-Schelvan et al. 2017). Therefore, LMP was evaluated in mexAM using digitonin extraction to evaluate cytosolic cathepsin B activity following 4 h treatment with NCZ, SIG, or AA nanoparticles. SIG, but not NCZ or AA, induced significant increases in cytoplasmic cathepsin B.

### Evaluation of NiO@Ni(OH)_2_ NP functionalization effect on inflammatory potential in mexAM

**Fig. 11.**
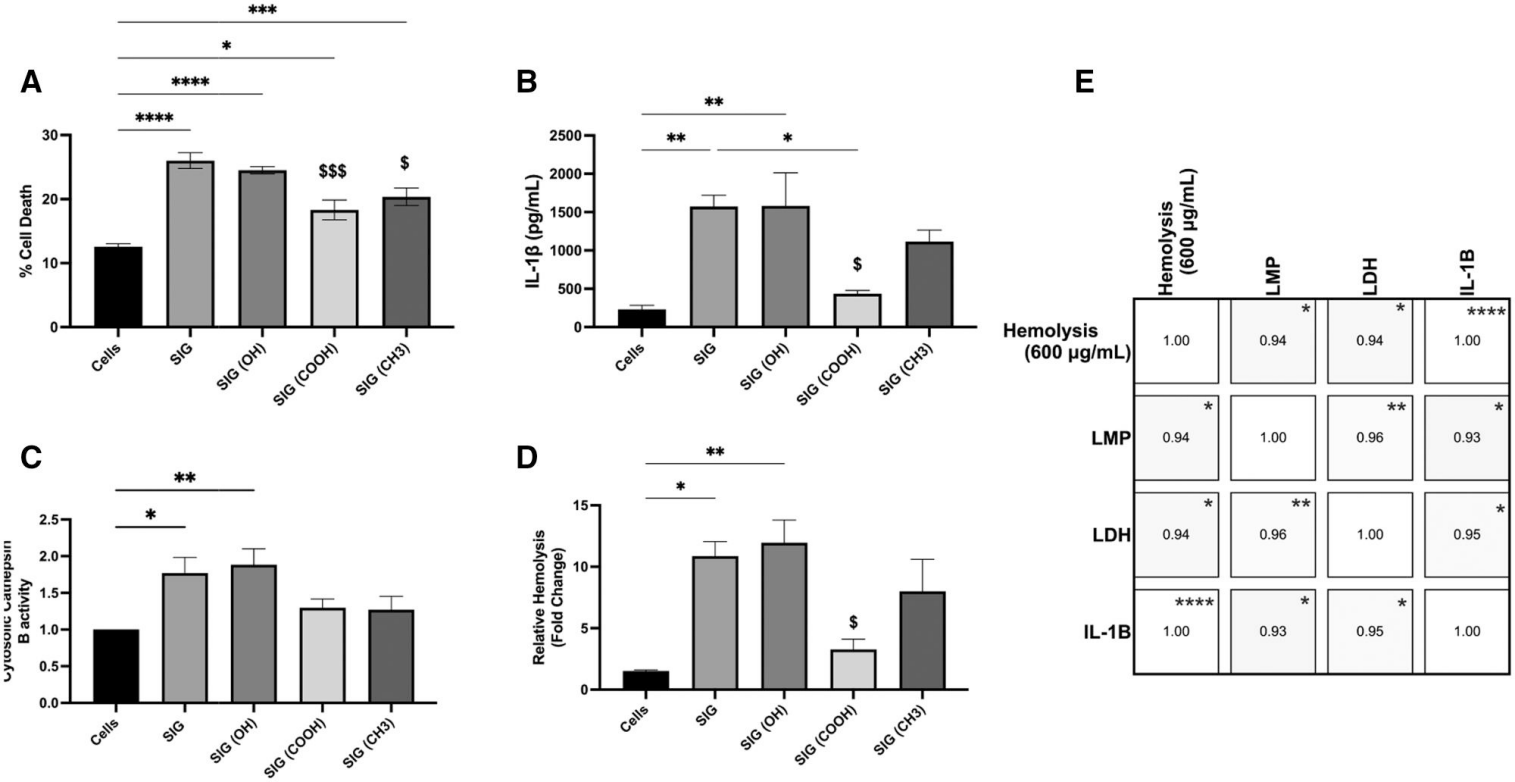
NiO@Ni(OH)2 NP LDH and IL-1β and LMP in mexAM macrophages. LPS-primed mexAM were cultured with SIG, SIG(–OH), SIG(–COOH), or SIG(–CH_3_) for 24 h and A) cell death and B) IL-1β release in supernatants was assessed. Naïve mexAM were treated with SIG, SIG(–OH), SIG(–COOH), or SIG(–CH_3_) for 4 h and C) LMP measured by cathepsin B activity in cytosolic extracts. Data expressed as mean ± SEM. *n* = 5. D) Hemolysis for 600 μg/ml dose of SIG as reported in Fig. 7. *n* = 3 experimental replicates. Asterisks indicate significance compared to control cells. Dollar sign symbols indicate significance to SIG Ni-X NP treated cells. One symbol: *P* < 0.05, 2 symbols: *P* < 0.01, 3 symbols: *P* < 0.001, and 4 symbols: *P* < 0.0001. E) Pearson correlation Hemolysis (600 μg/ml) vs LMP vs LDH vs IL-1β. Asterisk indicates significance to hemolysis. One symbol: *P* < 0.05, 2 symbols: *P* < 0.01, 3 symbols: *P* < 0.001, and 4 symbols: *P* < 0.0001.

Having observed that only raw SIG showed a significant increase in LMP, we evaluated the effect of functionalization on inflammatory potential in mexAM. LPS-primed mexAM were exposed to SIG (50 μg/ml), SIG(–OH), SIG(–COOH), or SIG(–CH_3_) for 24 h as before. Cell death was significantly increased above control for all Ni-X NP treatments (Fig. 11A); however, SIG(–COOH) and SIG(–CH_3_) showed a significant reduction in cell death compared with raw SIG. IL-1β release was significantly increased by SIG and SIG(–OH), whereas SIG(–COOH) and SIG(–CH_3_) were not significantly elevated (Fig. 11B). SIG(–COOH) significantly reduced IL-1β release compared with raw SIG. LMP was measured in mexAM treated with SIG (50 μg/ml), SIG(–OH), SIG(–COOH), or SIG(–CH_3_) for 4 h. SIG and SIG(–OH) caused a significant increase in cytosolic cathepsin B, but SIG(–COOH) and SIG(–CH_3_) were not significantly elevated above control cells (Fig. 11C). The cell death, IL-1β release, and LMP were compared with the hemolysis data for the 600 μg/ml 4 h exposure of SIG NP (Fig. 11D), and a similar trend was revealed. A Pearson correlation analysis revealed that Hemolysis–LMP had a correlation of 0.94, Hemolysis–LDH had a correlation of 0.94, and Hemolysis–IL-1β had a correlation of 1.00 at a *P < *0.05 significance level (Fig. 11E).

## Discussion

In this study, we investigated the effects of surface ligands on the biological activity of nickel compound nanoparticles (Ni-X NPs) and how they influence toxicity and inflammation in AMs while exploring how LMP contributed to the observed outcomes. We found that the biological activity of Ni-X NP is determined by a complex interplay between core composition, surface chemistry, Ni^2+^ release, and lysosomal interactions. Characterization revealed clear differences in particle morphology, crystallinity, and Ni^2+^ ion release for the Ni-X NP as described in Table 2. NCZ showed the highest crystallinity and minimal Ni^2+^ release, whereas AA had the lowest crystallinity and highest Ni^2+^ dissolution in SLF. SIG had intermediate features, including a mesoporous architecture and moderate Ni^2+^ ion release. These distinct differences in structural and chemical properties likely played a key role in the subsequent biological responses observed in RBC and macrophages in order to determine whether surface-ligand functionalization impacted inflammatory potential.

**Table 2.** Summary of results.

Nanoparticle (composition)	Particle size (avg nm)	Crystallinity	Hemolysis (RBC)	Uptake in AM (SSC increase)	Cell death in AM (LDH)	Cell viability in AM (MTS)	IL-1β in AM	Ni^2+^ release	**Cell death in mexAM** **(LDH)**	**IL-1β in** **mexAM**	LMP in mexAM (cathepsin B)
**NCZ (NiO)**	90	High	Mild at high dose	Moderate	None	Unaffected	Low, dose-dependent	Low	Low	Moderate	None
**SIG (NiO@Ni(OH)_2_)**	110	Low–moderate (mesoporous)	Strong	Highest	Minimal	Reduced, functionalization improved	High at low doses; functionalization reduced it	Moderate (reduced by functionalization)	Moderate	High	Significant
**AA (Ni@NiO@Ni(OH)_2_)**	12	None	Minimal	Moderate	Minimal	Strongly reduced, functionalization had no effect	High at low doses; minimal reduction w/functionalization	High (reduced by functionalization)	Low	Moderate	None

There was no pattern of change observed in the zeta potentials of functionalized Ni-X NP (as measured in media with 10% FBS). It is worth noting that the hemolysis assay used here did not include serum, which would have interfered with particle/membrane interaction necessary for hemolysis to occur. These conditions simulate the absence of the protein corona proposed in the acidic lysosomal environment. The protein corona encountered by the macrophage as it phagocytosis Ni-X and other nanoparticles is stripped off by lysosomal enzymes (Wang et al. 2013), suggesting that the protein corona is not a significant impactor of LMP.

NCZ, with its pure NiO composition, showed slightly more hemolysis than AA at the highest dose; however, it had the same effect on LMP in mexAM, causing similar cell death and IL-1β release in mexAM. Analysis of the change in median side scatter showed that NCZ had a smaller change than SIG; however, the difference in side scatter from particle to particle can be more indicative of their light scattering properties than a quantification of uptake, and future analyses should utilize additional methods to quantify any particle uptake differences that could exist between NCZ and SIG.

SIG with its NiO@Ni(OH)_2_ bilayer composition was unique in that –COOH functionalization (and –CH_3_ to a lesser degree) reduced hemolysis, LMP, cell death, and IL-1β release. This lack of effect with (–OH) functionalization may be due to the surface of SIG being highly decorated with –OH groups already, as seen in the FTIR characterization where modification did little to change its distinctive peaks, in contrast to –OH functionalization for the other Ni-X NP (Fig. 6B). The Ni(OH)_2_ shell present on this particular Ni-X NP is likely significant to its cytotoxicity and bears further scrutiny, as a comparison of pure NiO and Ni(OH)_2_ nanoparticles in A549 cells reported that Ni(OH)_2_ nanoparticles caused more cell death (Cambre et al. 2020). Furthermore, SIG was defined by a mesoporous structure, in contrast to the more crystalline structure of NCZ, which is notable as mesoporous nanoparticles have increased surface area due to the decoration of their surfaces with pores. As such, mesoporous materials could potentially have more membrane interactions than their more crystalline counterparts. Additionally, mesoporous NiO NP have been engineered for anti-tumor applications, as it has been reported that mesoporous NiO is degraded and releases Ni^2+^ in the acidic tumor microenvironment (Liu et al. 2018). Here, SIG, with its mesoporous structure, demonstrated a higher degree of dissolution in acidic lysosomal fluid than NCZ and initiated hemolysis in RBC and LMP in macrophages. Mesoporous silica nanoparticles are reported to cause less hemolysis than pristine silica nanoparticles (Slowing et al. 2009), which makes mesoporous silica nanoparticles useful for drug delivery in medicine. Despite the increase in surface area, mesoporous silica’s lack of membrane interactions is likely due to a disruption of the spacing of surface silanol groups (nearly free silanols) that have been implicated in silica’s binding to specific phospholipid headgroups and subsequent cytotoxicity (Pavan et al. 2022). Whether or not specific surface groups on the surface of NiX NP have specific phospholipid interactions remains to be discovered, but if those same surface groups exist on the surface of NCZ and SIG, it could explain how SIG has more membranolytic-based cytotoxicity that is disrupted by certain functional groups (e.g. –COOH). As such, both microporosity and interactions of metal oxide surface groups and phospholipids should be carefully considered in nanoparticle toxicology.

Despite the high dissolution of Ni^2+^ ions by AA, this Ni-X NP failed to initiate more than a minimal level of hemolysis. Although NiCl salt has been reported to cause hemolysis to increase 3-fold due to increased ROS, these studies used high NiCl concentrations (0.5 mM) with an incubation time of 24 h or more (Sharma et al. 2023, 2025). Given the lack of dose–response demonstrated by AA in the hemolysis assay, it seems unlikely that Ni^2+^ ions are driving hemolysis in this exposure model. Because AA caused a profound increase in cellular metabolism in AM and a significant increase in cell death in mexAM (while causing less IL-1β than SIG), there is reason to suspect that the cellular mechanisms that contribute to the bioactivity of the 2 Ni-X NPs are divergent despite the similarity of the @(Ni(OH)_2_) outer shell. The high Ni^2+^ dissolution and the small size of AA no doubt further contribute to differences in cellular mechanisms of bioactivity in macrophages. Additional studies are needed to understand these cellular mechanisms, as they seem insensitive to the effects of functionalization, despite functionalization reducing the Ni^2+^ dissolution of AA.

Taken together, these findings suggest that Ni-X NP toxicity and inflammation in macrophages are driven by a combination of particle composition, surface structure, lysosomal interactions, and ion release. SIG’s ability to cause cathepsin B release and hemolysis supports the hypothesis that LMP contributes to the pro-inflammatory response observed with this particular nanoparticle. Functionalization, particularly with –COOH or –CH_3_ groups, reduced inflammatory responses and Ni^2+^ release, and could be a potential strategy for mitigating nanoparticle-induced toxicity that results from LMP; however, they may not be as effective for nanoparticles that leach large amounts of ions easily, such as AA. Further studies are needed to explore in vivo relevance of functionalizations as well as long-term effects of Ni-X NP exposure. Additionally, the relevance of Ni^2+^ impact on mitochondrial function should be considered, and further analysis of the kinetics of LMP and Ni^2+^ interactions with the mitochondria following Ni-X NP exposures should be carefully evaluated.
